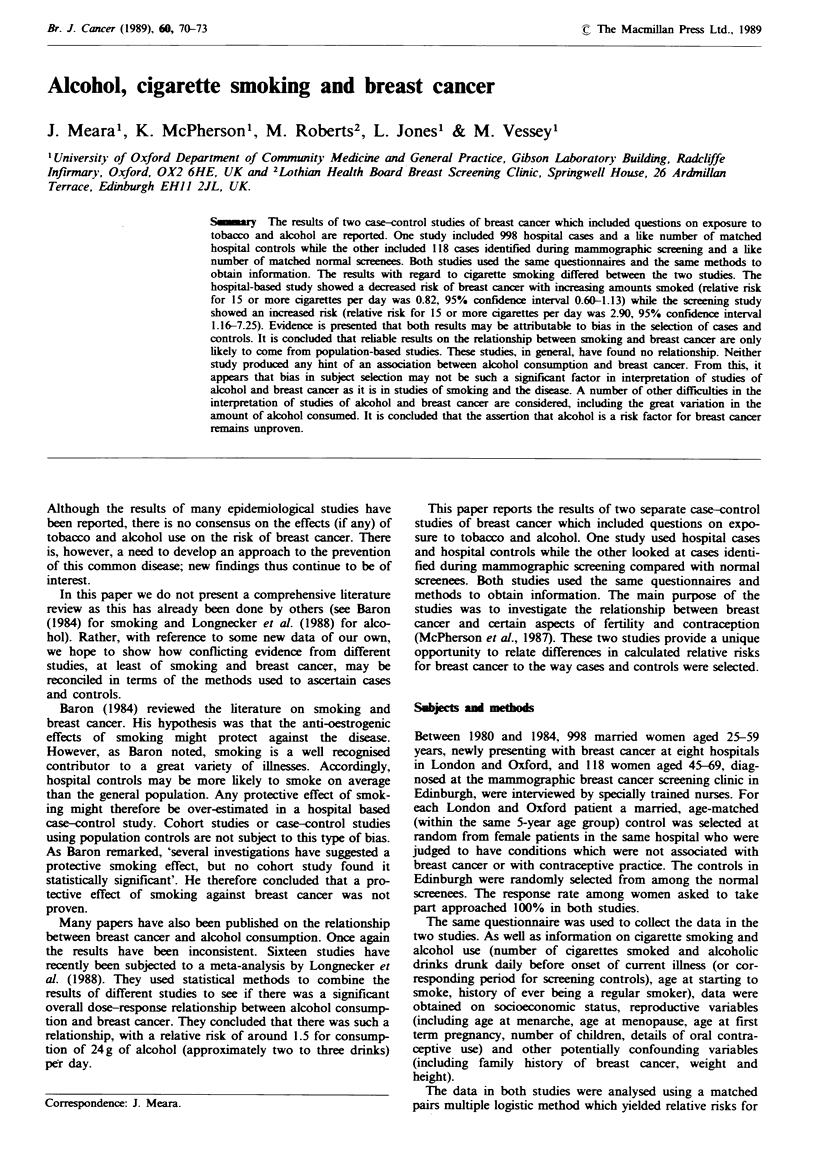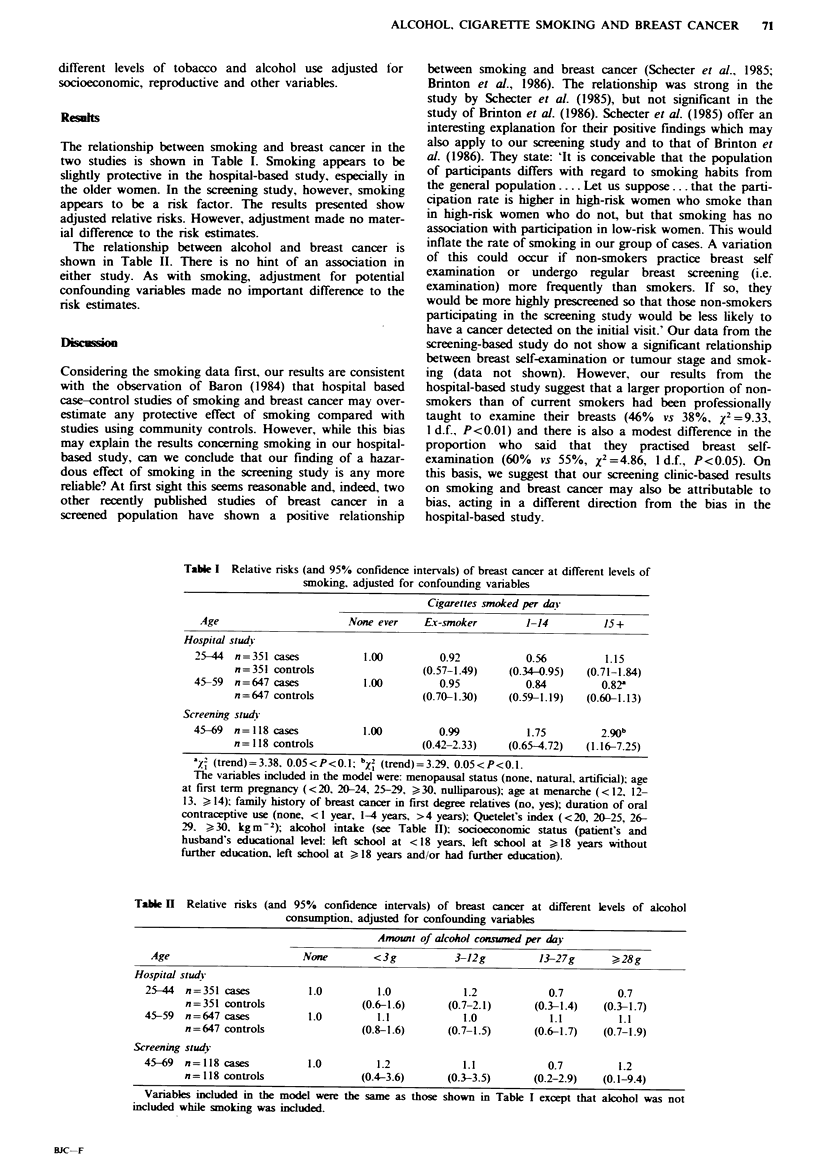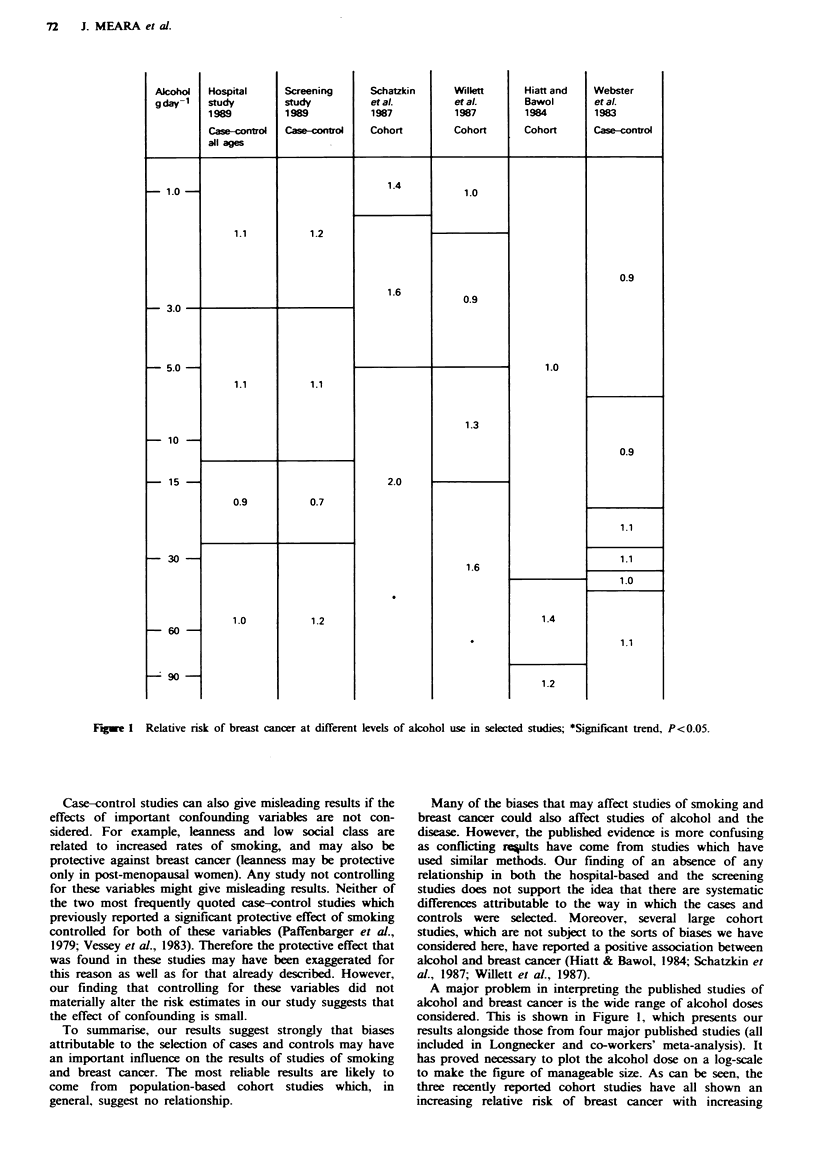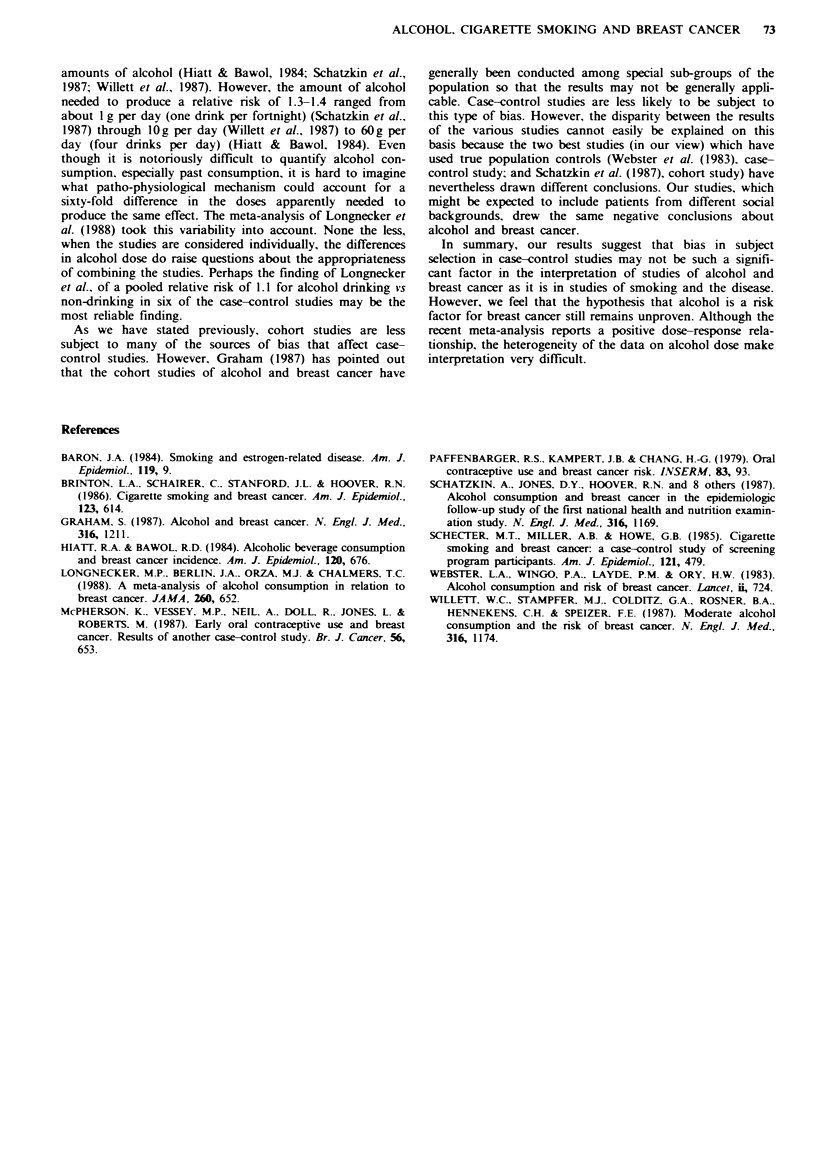# Alcohol, cigarette smoking and breast cancer.

**DOI:** 10.1038/bjc.1989.222

**Published:** 1989-07

**Authors:** J. Meara, K. McPherson, M. Roberts, L. Jones, M. Vessey

**Affiliations:** University of Oxford Department of Community Medicine and General Practice, Radcliffe Infirmary, UK.

## Abstract

The results of two case-control studies of breast cancer which included questions on exposure to tobacco and alcohol are reported. One study included 998 hospital cases and a like number of matched hospital controls while the other included 118 cases identified during mammographic screening and a like number of matched normal screenees. Both studies used the same questionnaires and the same methods to obtain information. The results with regard to cigarette smoking differed between the two studies. The hospital-based study showed a decreased risk of breast cancer with increasing amounts smoked (relative risk for 15 or more cigarettes per day was 0.82, 95% confidence interval 0.60-1.13) while the screening study showed an increased risk (relative risk for 15 or more cigarettes per day was 2.90, 95% confidence interval 1.16-7.25). Evidence is presented that both results may be attributable to bias in the selection of cases and controls. It is concluded that reliable results on the relationship between smoking and breast cancer are only likely to come from population-based studies. These studies, in general, have found no relationship. Neither study produced any hint of an association between alcohol consumption and breast cancer. From this, it appears that bias in subject selection may not be such a significant factor in interpretation of studies of alcohol and breast cancer as it is in studies of smoking and the disease. A number of other difficulties in the interpretation of studies of alcohol and breast cancer are considered, including the great variation in the amount of alcohol consumed. It is concluded that the assertion that alcohol is a risk factor for breast cancer remains unproven.


Br. J. Cancer (1989), 60, 70–73 © The Macmillan Press Ltd., 1989

### Alcohol, cigarette smoking and breast cancer

J. Meara[1], K. McPherson[1], M. Roberts[2], L. Jones[1] & M. Vessey[1]

[1]*University of Oxford Department of Community Medicine and General Practice, Gibson Laboratory Building, Radcliffe Infirmary, Oxford, OX2 6HE, UK and* [2]*Lothian Health Board Breast Screening Clinic, Springwell House, 26 Ardmillan Terrace, Edinburgh EH11 2JL, UK.*

**Summary** The results of two case–control studies of breast cancer which included questions on exposure to tobacco and alcohol are reported. One study included 998 hospital cases and a like number of matched hospital controls while the other included 118 cases identified during mammographic screening and a like number of matched normal screenees. Both studies used the same questionnaires and the same methods to obtain information. The results with regard to cigarette smoking differed between the two studies. The hospital-based study showed a decreased risk of breast cancer with increasing amounts smoked (relative risk for 15 or more cigarettes per day was 0.82, 95% confidence interval 0.60–1.13) while the screening study showed an increased risk (relative risk for 15 or more cigarettes per day was 2.90, 95% confidence interval 1.16–7.25). Evidence is presented that both results may be attributable to bias in the selection of cases and controls. It is concluded that reliable results on the relationship between smoking and breast cancer are only likely to come from population-based studies. These studies, in general, have found no relationship. Neither study produced any hint of an association between alcohol consumption and breast cancer. From this, it appears that bias in subject selection may not be such a significant factor in interpretation of studies of alcohol and breast cancer as it is in studies of smoking and the disease. A number of other difficulties in the interpretation of studies of alcohol and breast cancer are considered, including the great variation in the amount of alcohol consumed. It is concluded that the assertion that alcohol is a risk factor for breast cancer remains unproven.

Although the results of many epidemiological studies have been reported, there is no consensus on the effects (if any) of tobacco and alcohol use on the risk of breast cancer. There is, however, a need to develop an approach to the prevention of this common disease; new findings thus continue to be of interest.

In this paper we do not present a comprehensive literature review as this has already been done by others (see Baron (1984) for smoking and Longnecker *et al.* (1988) for alcohol). Rather, with reference to some new data of our own, we hope to show how conflicting evidence from different studies, at least of smoking and breast cancer, may be reconciled in terms of the methods used to ascertain cases and controls.

Baron (1984) reviewed the literature on smoking and breast cancer. His hypothesis was that the anti-oestrogenic effects of smoking might protect against the disease. However, as Baron noted, smoking is a well recognised contributor to a great variety of illnesses. Accordingly, hospital controls may be more likely to smoke on average than the general population. Any protective effect of smoking might therefore be over-estimated in a hospital based case–control study. Cohort studies or case–control studies using population controls are not subject to this type of bias. As Baron remarked, 'several investigations have suggested a protective smoking effect, but no cohort study found it statistically significant'. He therefore concluded that a protective effect of smoking against breast cancer was not proven.

Many papers have also been published on the relationship between breast cancer and alcohol consumption. Once again the results have been inconsistent. Sixteen studies have recently been subjected to a meta-analysis by Longnecker *et al.* (1988). They used statistical methods to combine the results of different studies to see if there was a significant overall dose–response relationship between alcohol consumption and breast cancer. They concluded that there was such a relationship, with a relative risk of around 1.5 for consumption of 24 g of alcohol (approximately two to three drinks) per day.

Correspondence: J. Meara.

This paper reports the results of two separate case–control studies of breast cancer which included questions on exposure to tobacco and alcohol. One study used hospital cases and hospital controls while the other looked at cases identified during mammographic screening compared with normal screenees. Both studies used the same questionnaires and methods to obtain information. The main purpose of the studies was to investigate the relationship between breast cancer and certain aspects of fertility and contraception (McPherson *et al.*, 1987). These two studies provide a unique opportunity to relate differences in calculated relative risks for breast cancer to the way cases and controls were selected.

### Subjects and methods

Between 1980 and 1984, 998 married women aged 25–59 years, newly presenting with breast cancer at eight hospitals in London and Oxford, and 118 women aged 45–69, diagnosed at the mammographic breast cancer screening clinic in Edinburgh, were interviewed by specially trained nurses. For each London and Oxford patient a married, age-matched (within the same 5-year age group) control was selected at random from female patients in the same hospital who were judged to have conditions which were not associated with breast cancer or with contraceptive practice. The controls in Edinburgh were randomly selected from among the normal screenees. The response rate among women asked to take part approached 100% in both studies.

The same questionnaire was used to collect the data in the two studies. As well as information on cigarette smoking and alcohol use (number of cigarettes smoked and alcoholic drinks drunk daily before onset of current illness (or corresponding period for screening controls), age at starting to smoke, history of ever being a regular smoker), data were obtained on socioeconomic status, reproductive variables (including age at menarche, age at menopause, age at first term pregnancy, number of children, details of oral contraceptive use) and other potentially confounding variables (including family history of breast cancer, weight and height).

The data in both studies were analysed using a matched pairs multiple logistic method which yielded relative risks for different levels of tobacco and alcohol use adjusted for socioeconomic, reproductive and other variables.

### Results

The relationship between smoking and breast cancer in the two studies is shown in Table I. Smoking appears to be slightly protective in the hospital-based study, especially in the older women. In the screening study, however, smoking appears to be a risk factor. The results presented show adjusted relative risks. However, adjustment made no material difference to the risk estimates.

The relationship between alcohol and breast cancer is shown in Table II. There is no hint of an association in either study. As with smoking, adjustment for potential confounding variables made no important difference to the risk estimates.

### Discussion

Considering the smoking data first, our results are consistent with the observation of Baron (1984) that hospital based case–control studies of smoking and breast cancer may overestimate any protective effect of smoking compared with studies using community controls. However, while this bias may explain the results concerning smoking in our hospital-based study, can we conclude that our finding of a hazardous effect of smoking in the screening study is any more reliable? At first sight this seems reasonable and, indeed, two other recently published studies of breast cancer in a screened population have shown a positive relationship between smoking and breast cancer (Schecter *et al*., 1985; Brinton *et al*., 1986). The relationship was strong in the study by Schecter *et al*. (1985), but not significant in the study of Brinton *et al*. (1986). Schecter *et al*. (1985) offer an interesting explanation for their positive findings which may also apply to our screening study and to that of Brinton *et al*. (1986). They state: 'It is conceivable that the population of participants differs with regard to smoking habits from the general population.... Let us suppose... that the participation rate is higher in high-risk women who smoke than in high-risk women who do not, but that smoking has no association with participation in low-risk women. This would inflate the rate of smoking in our group of cases. A variation of this could occur if non-smokers practice breast self examination or undergo regular breast screening (i.e. examination) more frequently than smokers. If so, they would be more highly prescreened so that those non-smokers participating in the screening study would be less likely to have a cancer detected on the initial visit.' Our data from the screening-based study do not show a significant relationship between breast self-examination or tumour stage and smoking (data not shown). However, our results from the hospital-based study suggest that a larger proportion of non-smokers than of current smokers had been professionally taught to examine their breasts (46% *vs* 38%, $\chi^2=9.33$, 1 d.f., $P<0.01$) and there is also a modest difference in the proportion who said that they practised breast self-examination (60% *vs* 55%, $\chi^2=4.86$, 1 d.f., $P<0.05$). On this basis, we suggest that our screening clinic-based results on smoking and breast cancer may also be attributable to bias, acting in a different direction from the bias in the hospital-based study.

**Table I** Relative risks (and 95% confidence intervals) of breast cancer at different levels of smoking, adjusted for confounding variables

| Age | | Cigarettes smoked per day | | | |
|---|---|---|---|---|---|
| | | None ever | Ex-smoker | 1–14 | 15+ |
| *Hospital study* | | | | | |
| 25–44 | $n=351$ cases | 1.00 | 0.92 | 0.56 | 1.15 |
| | $n=351$ controls | | (0.57–1.49) | (0.34–0.95) | (0.71–1.84) |
| 45–59 | $n=647$ cases | 1.00 | 0.95 | 0.84 | 0.82[a] |
| | $n=647$ controls | | (0.70–1.30) | (0.59–1.19) | (0.60–1.13) |
| *Screening study* | | | | | |
| 45–69 | $n=118$ cases | 1.00 | 0.99 | 1.75 | 2.90[b] |
| | $n=118$ controls | | (0.42–2.33) | (0.65–4.72) | (1.16–7.25) |

[a]$\chi_1^2$ (trend)=3.38, $0.05<P<0.1$; [b]$\chi_1^2$ (trend)=3.29, $0.05<P<0.1$.
The variables included in the model were: menopausal status (none, natural, artificial); age at first term pregnancy (<20, 20–24, 25–29, ≥30, nulliparous); age at menarche (<12, 12–13, ≥14); family history of breast cancer in first degree relatives (no, yes); duration of oral contraceptive use (none, <1 year, 1–4 years, >4 years); Quetelet's index (<20, 20–25, 26–29, ≥30, kg m$^{-2}$); alcohol intake (see Table II); socioeconomic status (patient's and husband's educational level: left school at <18 years, left school at ≥18 years without further education, left school at ≥18 years and/or had further education).

**Table II** Relative risks (and 95% confidence intervals) of breast cancer at different levels of alcohol consumption, adjusted for confounding variables

| Age | | Amount of alcohol consumed per day | | | | |
|---|---|---|---|---|---|---|
| | | None | <3 g | 3–12 g | 13–27 g | ≥28 g |
| *Hospital study* | | | | | | |
| 25–44 | $n=351$ cases | 1.0 | 1.0 | 1.2 | 0.7 | 0.7 |
| | $n=351$ controls | | (0.6–1.6) | (0.7–2.1) | (0.3–1.4) | (0.3–1.7) |
| 45–59 | $n=647$ cases | 1.0 | 1.1 | 1.0 | 1.1 | 1.1 |
| | $n=647$ controls | | (0.8–1.6) | (0.7–1.5) | (0.6–1.7) | (0.7–1.9) |
| *Screening study* | | | | | | |
| 45–69 | $n=118$ cases | 1.0 | 1.2 | 1.1 | 0.7 | 1.2 |
| | $n=118$ controls | | (0.4–3.6) | (0.3–3.5) | (0.2–2.9) | (0.1–9.4) |

Variables included in the model were the same as those shown in Table I except that alcohol was not included while smoking was included.

**Figure 1** Relative risk of breast cancer at different levels of alcohol use in selected studies; *Significant trend, $P<0.05$.

Case–control studies can also give misleading results if the effects of important confounding variables are not considered. For example, leanness and low social class are related to increased rates of smoking, and may also be protective against breast cancer (leanness may be protective only in post-menopausal women). Any study not controlling for these variables might give misleading results. Neither of the two most frequently quoted case–control studies which previously reported a significant protective effect of smoking controlled for both of these variables (Paffenbarger et al., 1979; Vessey et al., 1983). Therefore the protective effect that was found in these studies may have been exaggerated for this reason as well as for that already described. However, our finding that controlling for these variables did not materially alter the risk estimates in our study suggests that the effect of confounding is small.

To summarise, our results suggest strongly that biases attributable to the selection of cases and controls may have an important influence on the results of studies of smoking and breast cancer. The most reliable results are likely to come from population-based cohort studies which, in general, suggest no relationship.

Many of the biases that may affect studies of smoking and breast cancer could also affect studies of alcohol and the disease. However, the published evidence is more confusing as conflicting results have come from studies which have used similar methods. Our finding of an absence of any relationship in both the hospital-based and the screening studies does not support the idea that there are systematic differences attributable to the way in which the cases and controls were selected. Moreover, several large cohort studies, which are not subject to the sorts of biases we have considered here, have reported a positive association between alcohol and breast cancer (Hiatt & Bawol, 1984; Schatzkin et al., 1987; Willett et al., 1987).

A major problem in interpreting the published studies of alcohol and breast cancer is the wide range of alcohol doses considered. This is shown in Figure 1, which presents our results alongside those from four major published studies (all included in Longnecker and co-workers' meta-analysis). It has proved necessary to plot the alcohol dose on a log-scale to make the figure of manageable size. As can be seen, the three recently reported cohort studies have all shown an increasing relative risk of breast cancer with increasing amounts of alcohol (Hiatt & Bawol, 1984; Schatzkin *et al.*, 1987; Willett *et al.*, 1987). However, the amount of alcohol needed to produce a relative risk of 1.3–1.4 ranged from about 1 g per day (one drink per fortnight) (Schatzkin *et al.*, 1987) through 10 g per day (Willett *et al.*, 1987) to 60 g per day (four drinks per day) (Hiatt & Bawol, 1984). Even though it is notoriously difficult to quantify alcohol consumption, especially past consumption, it is hard to imagine what patho-physiological mechanism could account for a sixty-fold difference in the doses apparently needed to produce the same effect. The meta-analysis of Longnecker *et al.* (1988) took this variability into account. None the less, when the studies are considered individually, the differences in alcohol dose do raise questions about the appropriateness of combining the studies. Perhaps the finding of Longnecker *et al.*, of a pooled relative risk of 1.1 for alcohol drinking *vs* non-drinking in six of the case–control studies may be the most reliable finding.

As we have stated previously, cohort studies are less subject to many of the sources of bias that affect case–control studies. However, Graham (1987) has pointed out that the cohort studies of alcohol and breast cancer have generally been conducted among special sub-groups of the population so that the results may not be generally applicable. Case–control studies are less likely to be subject to this type of bias. However, the disparity between the results of the various studies cannot easily be explained on this basis because the two best studies (in our view) which have used true population controls (Webster *et al.* (1983), case–control study; and Schatzkin *et al.* (1987), cohort study) have nevertheless drawn different conclusions. Our studies, which might be expected to include patients from different social backgrounds, drew the same negative conclusions about alcohol and breast cancer.

In summary, our results suggest that bias in subject selection in case–control studies may not be such a significant factor in the interpretation of studies of alcohol and breast cancer as it is in studies of smoking and the disease. However, we feel that the hypothesis that alcohol is a risk factor for breast cancer still remains unproven. Although the recent meta-analysis reports a positive dose–response relationship, the heterogeneity of the data on alcohol dose make interpretation very difficult.

### References

BARON, J.A. (1984). Smoking and estrogen-related disease. *Am. J. Epidemiol.*, **119**, 9.

BRINTON, L.A., SCHAIRER, C., STANFORD, J.L. & HOOVER, R.N. (1986). Cigarette smoking and breast cancer. *Am. J. Epidemiol.*, **123**, 614.

GRAHAM, S. (1987). Alcohol and breast cancer. *N. Engl. J. Med.*, **316**, 1211.

HIATT, R.A. & BAWOL, R.D. (1984). Alcoholic beverage consumption and breast cancer incidence. *Am. J. Epidemiol.*, **120**, 676.

LONGNECKER, M.P., BERLIN, J.A., ORZA, M.J. & CHALMERS, T.C. (1988). A meta-analysis of alcohol consumption in relation to breast cancer. *JAMA*, **260**, 652.

McPHERSON, K., VESSEY, M.P., NEIL, A., DOLL, R., JONES, L. & ROBERTS, M. (1987). Early oral contraceptive use and breast cancer. Results of another case–control study. *Br. J. Cancer*, **56**, 653.

PAFFENBARGER, R.S., KAMPERT, J.B. & CHANG, H.-G. (1979). Oral contraceptive use and breast cancer risk. *INSERM*, **83**, 93.

SCHATZKIN, A., JONES, D.Y., HOOVER, R.N. and 8 others (1987). Alcohol consumption and breast cancer in the epidemiologic follow-up study of the first national health and nutrition examination study. *N. Engl. J. Med.*, **316**, 1169.

SCHECTER, M.T., MILLER, A.B. & HOWE, G.B. (1985). Cigarette smoking and breast cancer: a case–control study of screening program participants. *Am. J. Epidemiol.*, **121**, 479.

WEBSTER, L.A., WINGO, P.A., LAYDE, P.M. & ORY, H.W. (1983). Alcohol consumption and risk of breast cancer. *Lancet*, **ii**, 724.

WILLETT, W.C., STAMPFER, M.J., COLDITZ, G.A., ROSNER, B.A., HENNEKENS, C.H. & SPEIZER, F.E. (1987). Moderate alcohol consumption and the risk of breast cancer. *N. Engl. J. Med.*, **316**, 1174.